# Superior Creep Resistance and Remnant Strength of Novel Tempered Ferritic-Martensitic Steels Designed by Element Addition

**DOI:** 10.3390/ma15093327

**Published:** 2022-05-06

**Authors:** Hang Wang, Keer Li, Wei Chen, Lihong Han, Yaorong Feng

**Affiliations:** 1 College of Mechanical Engineering, Xi’an Shiyou University, Xi’an 710065, China; wanghang008@cnpc.com.cn; 2 State Key Laboratory of Performance and Structural Safety for Petroleum Tubular Goods and Equipment Materials, CNPC Tubular Goods Research Institute, Xi’an 710077, China; hanlh@cnpc.com.cn (L.H.); fengyaorong@cnpc.com.cn (Y.F.); 3 State Key Laboratory for Mechanical Behavior of Materials, Xi’an Jiaotong University, Xi’an 710049, China; lke3119302137@stu.xjtu.edu.cn

**Keywords:** steels, creep, strength, microstructures, carbides

## Abstract

The in situ combustion (ISC) technique is promisingly applied in heavy oil recovery, whereas the operation inevitably causes high temperature and high pressure for a long duration in the thermal recovery well. As a critical component, oil casing, traditionally made of plain carbon steel in China, generally suffers from poor creep resistance and degraded remnant strength under such a harsh environment, which leads to frequent casing damage and inferior recovery efficiency. In this study, a strategy was adopted to tackle the issue by adding chromium (Cr) element into the plain carbon steel. We designed two types of novel steel with the respective addition of 1 wt.% and 13 wt.% Cr element into plain carbon steel for oil casing. Surprisingly, the trace addition of Cr element with 1 wt.% effectively lowered the creep rate in a creep test at 600 °C and 400 MPa and maintained high remnant tensile strength after creep. More significantly, prior creep history dramatically enhanced remnant strength when Cr element was added up to 13 wt.%. After a long-term creep time of 96 h, the samples were conferred by a stress increment of ~92.5 MPa (~11.0%) relative to the creep-free counterparts, whereas the value was reduced by ~158.4 MPa (~17.8%) for plain carbon steel under the same deformation conditions. Such superior mechanical performances in the Cr-doped steels are mainly ascribed to precipitation retardation of carbides and sluggish precipitate coarsening, which continuously favors a precipitation–strengthening effect in steel. These findings provide a fundamental understanding of precipitation response and creep behaviors and, more importantly, enable the development of high-performance steels used in the field of unconventional petroleum and gas resources.

## 1. Introduction

Heavy oil accounts for approximately 53% of total world reserves, and thermal recovery is generally used to enhance oil recovery during exploitation [1]. Compared with other thermal recovery methods, in situ combustion (ISC) is a promising technique, owing to higher fuel efficient [1,2,3,4,5]. ISC is performed, the fire front moves forward to heat heavy oil and thereby constructs a harsh environment of high temperature and high pressure [1]. As a critical component in the thermal recovery well, oil casing inevitably suffers from an elevated temperature of up to ~600 °C and a concurrent complex stress loading of ~400 MPa for a duration time up to 96 h [6]. Currently, oil casing is conventionally machined by plain carbon steels in China due to easy acquisition and moderate cost [6]. The typical failure mode encountered by the material in heating-loading environment involves creep deformation and fracture, which leads to frequent casing damage. This drawback degrades recovery efficiency and in turn increases economic cost. Therefore, enhancement of creep resistance is an urgent requirement for the usage of oil casing in heavy oil thermal wells.

Creep mechanisms invoked in engineering materials have been disclosed to involve non-conservative vacancy-assisted dislocation climb over obstacles, thermally activated cross-slip, solution-induced viscous drag on dislocations, jog-assisted dislocation motion, movement through dislocation intersections, and grain boundary sliding [7,8,9,10,11]. Such insights prompt researchers to develop creep-resistant alloys via obstruction of the dislocation motion in the parent matrix. Chen et al. designed polysynthetic nano-twinned TiAl single crystals that possessed creep lifetime and minimum creep rates superior to those for commercial 4822 TiAl polycrystalline material by more than one order of magnitude [12]. Gao et al. designed a segregation-sandwiched stable interface to suffocate nanoprecipitate coarsening through the addition of Sc element, enabling a dramatic reduction in creep rate in Al-Cu alloys [13]. As for e steels, one striking microstructural feature is that the soft matrix is embedded by intermetallic precipitates. Thermodynamical stability of the precipitates at elevated temperature thus becomes a crucial consideration. In this regard, high creep resistance can be conferred supposing that the precipitates have a retarded morphologic evolution and a slow coarsening kinetics. Yamamoto et al. uncovered that the addition of aluminum element permitted stabilization of an austenitic structure by means of the formation of Al_2_O_3_ and rendered steels with a creep-rupture lifetime in excess of 200 h at 750 °C and 100 MPa in air [14]. Another representative example is 18Ni maraging steel, which is usually applied in turbine engines at high operating temperatures. The formation of a *β*-NiAl ordered phase and (Fe, Cr)_2_(W, Mo) a Laves phase endows anomalous creep resistance due to high thermodynamic stability of the precipitates [15]. Taking the above together, we surmise that thermodynamically and mechanically stabilizing precipitates is perhaps a feasible strategy to enhance the creep resistance of the plain carbon steel currently used in oil casing.

Considerable investigations have focused on the effect of alloying elements on creep properties of steel [16,17,18,19,20]. One vital element is chromium (Cr), which is a kind of the stabilization element of the ferrite phase [21]. Abe et al. demonstrated that the creep strength of low-carbon ferrite steels was progressively increased with an increase in Cr content [22]. Recent research concerning the improved creep properties in 12Cr tempered ferritic-martensitic steels has revealed three types of precipitates, including M_23_C_6_, VX, and Laves phase, in microstructures [23]. Such research has demonstrated the capability of Cr-addition to enhance the creep resistance of steel. This issue deserves to be further explored in steels currently used under the specific temperature-loading conditions.

Tensile properties are usually considered the primary mechanical indicators for the design of oil casing string [24,25]. Therefore, it is of great concern to assess the effect of high-temperature creep on remnant tensile strength in order to ensure continuous reliable service of oil casing. Recent research has demonstrated that tensile properties of medium- and high-alloy steels are significantly altered by prior creep–fatigue interaction or prior low-cycle fatigue [26,27,28,29]. Nevertheless, relevant studies regarding the influence of prior creep history on remnant tensile properties of steel are scarce so far.

Motivated by the above issues, in this study, efforts were dedicated to design novel low-alloy steels with Cr addition in order to improve creep resistance and subsequent remnant tensile strength. It was revealed that the addition of Cr element effectively postpones creep damage and enhances remnant tensile strength. This superior mechanical performance is mainly ascribed to precipitation retardation of carbides and sluggish precipitate coarsening, which continuously contributes to precipitation strengthening of the steels. These findings provide a fundamental understanding of precipitation response and creep behaviors and offer guidelines for the development of superior creep-resistant steels for use in the petroleum industry.

## 2. Experimental Procedures

To investigate the effect of Cr-addition on high-temperature creep resistance of plain carbon steels, two types of steels with nominal Cr addition of 1% and 13% in weight percent were designed together with the plain carbon steel without Cr addition for comparison. The actual chemical compositions of the three kinds of steels are listed in Table 1. For the sake of convenience, hereafter, they are separately referred to as 0Cr, 1Cr and 13Cr steels on the basis of Cr content. These steels supplied by Baoshan Iron and Steel Co., Ltd. (Shanghai, China) were first austenitized at 900 °C for 0.5 h, followed by water quenching to ambient temperature. Subsequent tempering was performed at 760 °C for 3 h, followed by air cooling. 

Creep samples with a gauge diameter of 5 mm and a gauge length of 25 mm were fabricated following the ASTEM E8/E8M-15a standard. Interrupted creep tests were implemented at 600 °C and 400 MPa on a Gleeble 3500 thermal simulator. Five interrupted loading durations were set as 12, 24, 48, 72 and 96 h. The temperature deviation during the testing period was less than 3 °C. After creep testing, uniaxial tensile tests were conducted using an MTS880 material testing machine at a constant strain rate of 6.67 × 10^−4^ s^−1^ at ambient temperature. At least five samples were tested under each condition to ensure the reproducibility of the results.

Metallographic analysis was carried out on an Olympus PMG3 optical microscope (OM). The samples were etched in a solution containing 20 mL nitric acid (60% concentration), 20 mL hydrochloric acid (36% concentration) and 60 mL water. For transmission electron microscopic (TEM) analyses, discs with a diameter of 3 mm were punched from the samples and mechanically ground to a thickness of 60 μm. The discs were further thinned by two-jet electropolishing in a mixed solution of 5% perchloric acid and 95% ethanol. TEM observations were conducted on a JEOL-2100F microscope operating at 200 kV. To acquire the composition information of carbides, analysis of chemical elements was conducted using an energy-dispersive X-ray spectrometer (EDS) attached to a TEM microscope in high-angle annular dark field (HAADF) mode.

## 3. Results

### 3.1. Creep Properties

**Figure 1:**
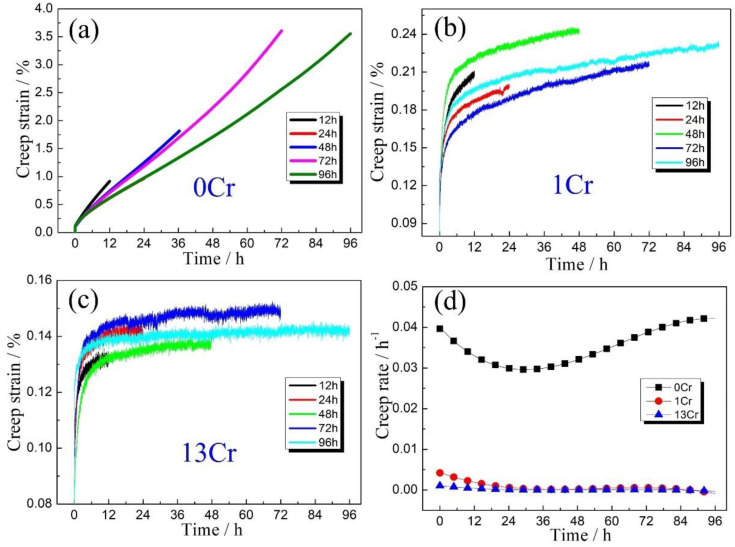
Typical creep curves of three types of samples subjected to various creep times: (**a**) 0Cr samples; (**b**) 1Cr samples and (**c**) 13Cr samples. (**d**) Corresponding variations of steady-state creep rate against creep time.

Interrupted creep tests were performed for various duration periods on three types of Cr-content samples at an elevated temperature of 600 °C and tensile loading of 400 MPa. Corresponding creep strain (*ε*)–time (*t*) curves are shown in Figure 1. A general feature is that creep strain continuously increases with prolonged duration in all three types of samples, whereas creep deformation enters into a steady-state creep stage after shortly residing at the initial transient creep stage, although at the shortest duration time of 12 h. For 0Cr samples, *ε* was measured as ~0.92% after creeping for 12 h, whereas the value rose to ~3.56% when the creep time was increased to 96 h (Figure 1a). For the 1Cr samples, the creep strains were ~0.20% and 0.23% for two duration periods, as shown in Figure 1b. It is apparent that creep strain decreased at the same duration time when introducing Cr addition into the plain carbon steels. Such a suppression effect of creep strain became much more remarkable when the addition of Cr element was further increased to 13 wt.% (Figure 1c). The creep strain, *ε*, sharply descended to ~0.12% at a duration time of 12 h, and the value is increased to ~0.14%, although the creep time was prolonged to 96 h. Moreover, upon careful examination of the *ε*-*t* curves in Figure 1a–c, it can be found that the steady-state creep stage becomes flattened relative to the horizontal coordinate axis with the addition of Cr content. This trend was further quantified using creep rate, $\dot{\varepsilon }$, which is determined by differentiating creep strain, *ε*, with respect to creep time, *t*. The corresponding variations in $\dot{\varepsilon }$ with *t* for the three types of samples are presented in Figure 1d. Obviously, both 1Cr and 13Cr samples always maintain a stable creep rate, $\dot{\varepsilon }$, with values around ~3.14 × 10^−4^ h^−1^ and ~2.59 × 10^−5^ h^−1^, respectively, which is considerably lower than that of 0Cr samples (~2.98 × 10^−2^ h^−1^). This result strongly suggests that the addition of Cr element endows the steels with pronounced creep resistance.

### 3.2. Tensile Properties after Prior Creep

**Figure 2:**
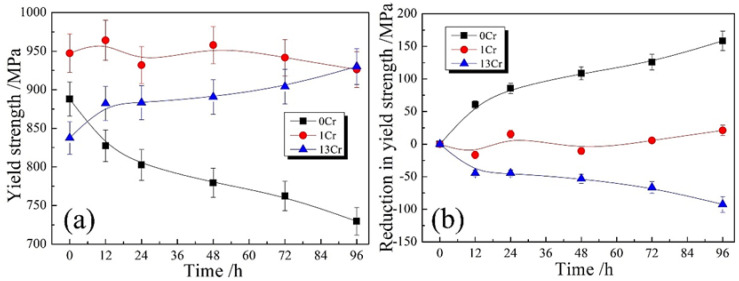
Tensile properties of three types of as-crept samples: (**a**) variations in yield strength with creep time; (**b**) variations in reduction in yield strength with creep time. Reduction in yield strength is defined as the difference of yield strength between the creep-free samples and the as-crept samples.

The following tensile testing was performed on the as-crept samples, and the resulting yield strength, $\sigma_{y}$, was extracted from tensile stress–strain curves. Figure 2a shows the variations in yield strength, $\sigma_{y}$, with creep time, *t*, for the three types of samples. It is seen that $\sigma_{y}$ is continuously decayed in the 0Cr samples with prolonged creep time, *t*. $\sigma_{y}$ was measured as ~890 MPa in the initial tempered state, whereas the value declined to ~830 MPa when creep was performed for 12 h and, finally, to ~730 MPa after the creep time was prolonged to 96 h. When 1 wt.%Cr was added into the steels, the dependence of $\sigma_{y}$ on creep time was dramatically changed. $\sigma_{y}$ was approximately 940 MPa in the creep-free sample, whereas the value was slightly reduced to ~930 MPa after a creep time of 96 h. This means that the 1Cr samples almost maintained a constant $\sigma_{y}$ in the range of the creep time, whereas prior creep history seems to have a negligible detrimental effect on remnant tensile strength. For the 13Cr samples, the other scenario appears in the $\sigma_{y}$–*t* curve. In the creep-free sample, $\sigma_{y}$ was measured as ~840 MPa, whereas the value sharply increased with prolonged creep time, ascending from ~880 MPa at 12 h to ~930 MPa at 96 h. It is surprising that the creep history enhances remnant tensile strength in the 13Cr samples rather than conventionally deteriorating the property as in the 0Cr samples. Such strengthening effect of Cr addition on remnant tensile strength is further characterized by the curves of reduction in yield strength, $\Delta \sigma_{y}$, and creep time, *t* (Figure 2b). Here, the yield strength, $\sigma_{y}$, in the creep-free sample was chosen as a base point, and the difference with the $\sigma_{y}$ after a given creep time is defined as $\Delta \sigma_{y}$. It is apparent that the Cr addition markedly reversed the evolution trend of yield strength, $\sigma_{y}$, with increased addition of Cr element. The samples can even be strengthened after suffering from a period of creep deformation when they have Cr content of 13 wt.%. 

### 3.3. Optical Morphologies after Creep

**Figure 3:**
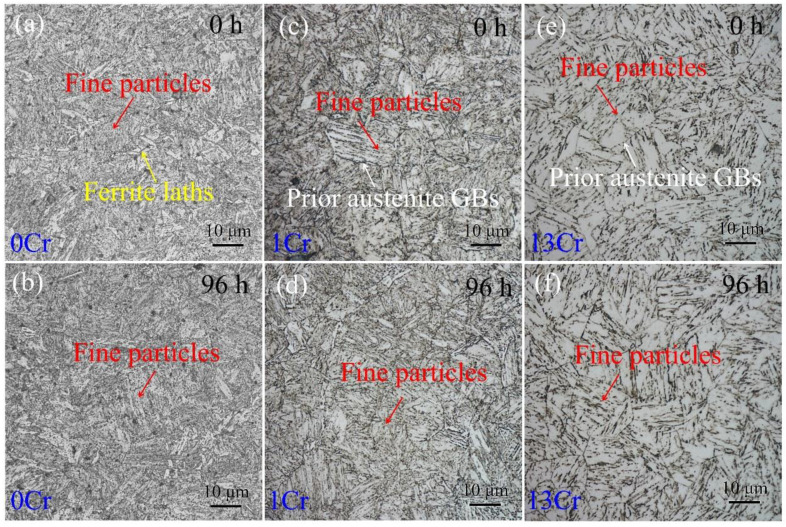
Optical morphologies of three types of samples after creeping for various times: (**a**,**b**) 0Cr samples; (**c**,**d**) 1Cr samples; and (**e**,**f**) 13Cr samples. (**a**,**c**,**e**) Creep-free samples; (**b**,**d**,**f**) samples crept for 96 h.

Figure 3 shows optical morphologies of the samples crept for various times. For the 0Cr samples without creeping (or the tempered state), martensitic laths produced by water quenching mostly disappeared, and instead, a large number of fine particles in the dot contrast dispersed in the microstructure (Figure 3a). When the samples encountered creeping, the number of fine particles decreased. Figure 3b,c shows optical morphology of the creep-free 1Cr sample. Unlike the 0Cr samples, prior austenite grains with an average size of 20 μm were detected, and fine particles were found to be distributed within the austenite grain interiors. Upon careful examination, the number of the particles was less than that in the 0Cr samples, and several matrix areas were not decorated by the particles. Additionally, these particles were aligned in a certain direction as opposed to random distribution. Figure 3d shows optical morphology of the 1 Cr sample after creeping for 96 h. Compared with Figure 3c, there is no apparent microstructural change, irrespective of prior austenite boundaries and the internal particle distribution. Regarding the 13Cr samples, one striking microstructural feature is that the number of the particles is notably decreased so that extensive space is left in bright contrast, as shown in Figure 3e. Such microstructural morphology is maintained, although the samples suffer from a long-term creep of 96 h (Figure 3f).

### 3.4. TEM Microstructures after Creep

**Figure 4:**
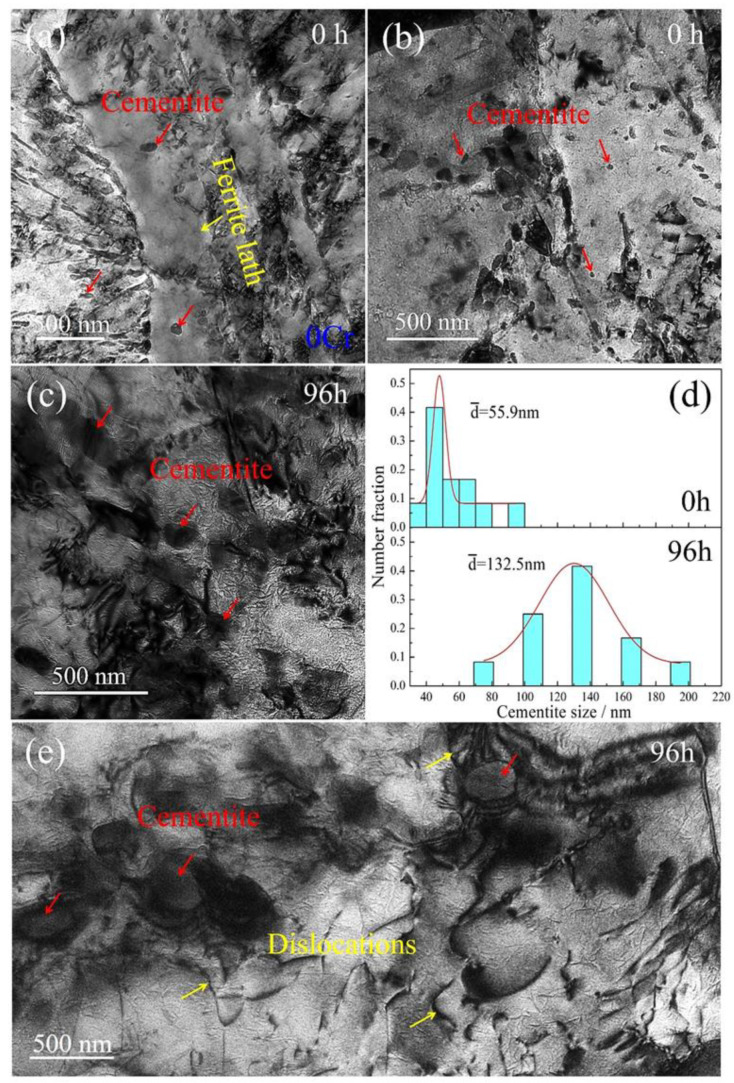
TEM images of the 0Cr samples after various creep times: (**a**,**b**) 0 h; (**c**) 96 h. Here, (**a**) is overall morphology, whereas (**b**,**c**) are high-magnification morphologies. (**d**) Statistical size distributions of the cementite particles after creeping for the two durations; (**e**) TEM image of cementite particles surrounded by dislocations.

To investigate the creep properties and remnant tensile strength caused by the addition of Cr element in the samples, the crept microstructures were characterized using TEM microscopy. Figure 4 shows TEM images of the 0Cr samples after various creep times. When the samples do not undergo creep loading, i.e., they are in the tempered state; the microstructure is composed of typical tempered sorbite, where the quenching martensite laths are decomposed into a dual-phase structure of ferrite and cementite (Figure 4a). The ferrite maintains the lath morphology of martensite with a thickness of ~1.5 μm, whereas the cementite disperses either along ferrite boundaries or inside the ferrite interiors. Such particle distribution is consistent with the dense dot contrast in the corresponding optical image in Figure 3a. Figure 4b is a high-magnification TEM image of the cementite particles in the 0 h sample. The cementite particles in an ellipsoid shape are distributed in the microstructure. When suffering from creep deformation, the cementite particles are coarsened. Figure 4c shows TEM morphology at a creep time of 96 h. It is apparent that the numerical density of the cementite is decreased, and the corresponding dimensions are increased dramatically. To further quantitatively characterize the coarsening, statistical measurement of the cementite particles was conducted after extensive TEM observations. Figure 4d shows size distribution of the cementite particles as a function of creep time. The particle sizes in the 0 h samples are in a range of 35–95 nm, with an average value of ~55.9 nm, whereas the sizes are sharply increased to between 75 and 195 nm, with an average value of ~132.5 nm, when the creep time is prolonged to 96 h. This coarsening indicates that the cementite particles in plain carbon steels are, in essence, microstructurally unstable when suffering from creep loading. In addition to cementite particles, dislocations can be frequently found in the microstructure. Figure 4e shows a TEM image of cementite particles surrounded by dislocations in the 0Cr sample crept for 96 h. A large number of dislocations are distributed in the ferrite parent, and they are piled-up in front of cementite particles. It can be asserted that these dislocations result from creep strain upon loading. Given quick coarsening of cementite particles during the creep duration, their interaction with cementite should promote the coarsening of cementite.

**Figure 5:**
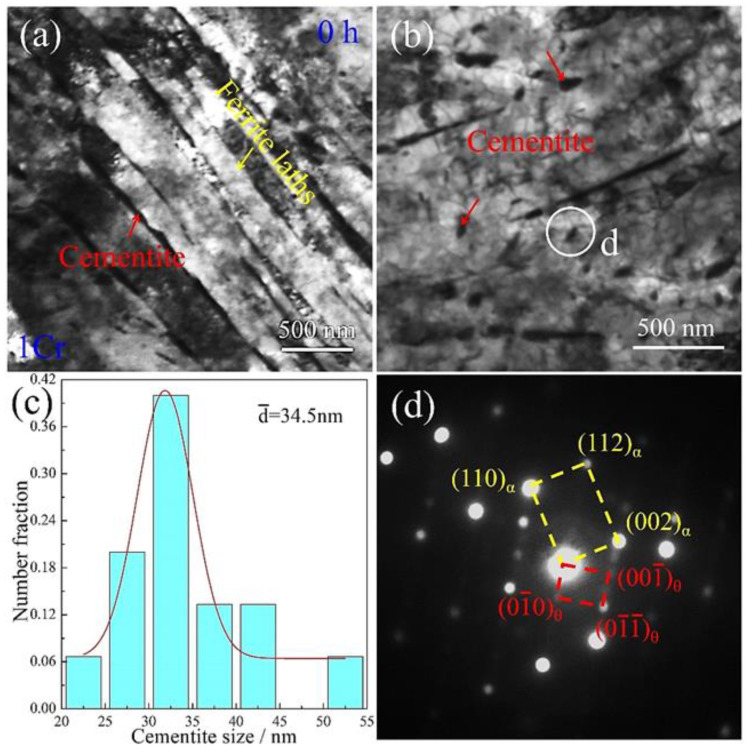
TEM images of the 1Cr creep-free samples: (**a**) BF image showing ferrite laths and cementite along the ferrite lath boundaries; (**b**) BF image showing cementite in ferrite lath interiors; (**c**) statistical size distribution of cementite particles; (**d**) SAED pattern taken from the circle in (**b**) under the ${\left[1\bar{1}0\right]}_{\alpha }$ zone axis.

Figure 5 shows TEM microstructural morphologies of the 1Cr creep-free samples. A bundle of ferrite laths transverses across the micrography from the upper left to the lower right, as shown in Figure 5a. The lath thickness was measured as approximately 300 nm. Some elongated rods line up along ferrite lath boundaries. These rods were further ascertained to be the alloyed cementite by chemical composition analysis, as demonstrated in Section 3.5 below. Such distribution features of the cementite correlate with the optical observations in Figure 3c. In addition to along the ferrite boundaries, cementite was also found to be nucleated in the ferrite lath interiors (Figure 5b.) The particle morphology and the particle dimensions are fairly small. Statistical measurements in Figure 5c manifest that the dimensions of the cementite are in a range of 24–50 nm, with an average value of ~34.5 nm. Furthermore, the crystallographic orientation relationship between the cementite and the ferrite matrix was characterized. Figure 5d shows corresponding selected area electron diffraction (SAED) pattern under the ${\left[1\bar{1}0\right]}_{\alpha }$ zone axis. The crystallographic orientation relationship between the two phases was determined to be ${\left\{001\right\}}_{\alpha }//{\left\{011\right\}}_{\theta }$ and ${\langle 110\rangle }_{a}//{\langle 100\rangle }_{\theta }$.

**Figure 6:**
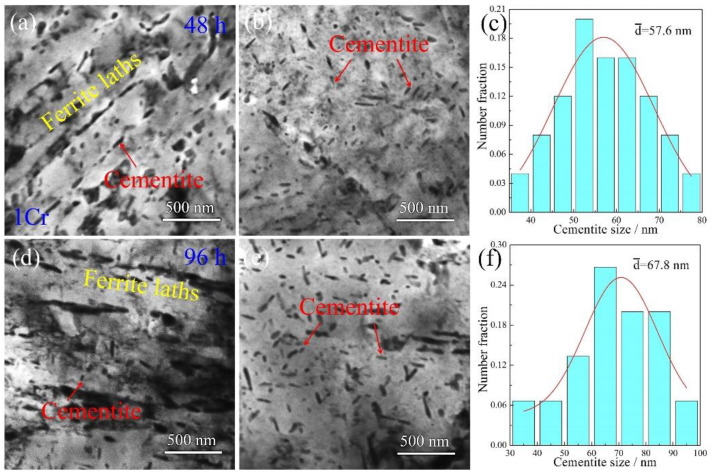
TEM images of the 1Cr samples crept for various durations: (**a**–**c**) 48 h; (**d**–**f**) 96 h. (**a**,**d**) BF images showing ferrite laths and cementite rods along ferrite lath boundaries; (**b**,**e**) BF image showing cementite particles in ferrite lath interiors; (**c**,**f**) statistical size distribution of cementite particles.

TEM images of the 1Cr samples undergoing creep deformation for various durations are presented in Figure 6. When the samples are crept for 48 h, cementite still presents two types of morphologies in the microstructure (Figure 6a). The cementite rods line up along ferrite boundaries to enclose the ferrite laths, whereas their dimensions are not obviously changed in comparison to those in the 1Cr creep-free samples. The cementite particles in ferrite interiors, however, are marginally coarsened together with the increase in numerical density. This microstructural variation is much more apparent in the thick ferrite lath interior (Figure 6b). Furthermore, cementite particles have a tendency to become elongated rods. Figure 6c shows corresponding statistical measurements of cementite size. Given different aspect ratios among the cementite rods, only the rod thickness was measured to represent cementite size. As a result, the values range from 34 nm to 78 nm, with an average value of around 57.6 nm. Figure 6d shows TEM morphology of the 1Cr sample after creeping for 96 h. Tangled dislocations are developed in the ferrite lath interior. These dislocations should result from accumulative strain after the long-term creep time. Figure 6e shows TEM micrography of cementite in a thick ferrite lath. The aspect ratios of the cementite rods are increased, and the morphology of elongated rods becomes more obvious. Statistical measurements show that the rod thickness is in a range of 36–92 nm, with an average value of ~67.8 nm (Figure 5f). Apparently, the increment of the cementite size is small in comparison to that in the 48 h crept samples (~57.6 nm). This indicates that the addition of 1 wt.% Cr element stabilizes cementite structure and retards particle coarsening to a considerable extent.

**Figure 7:**
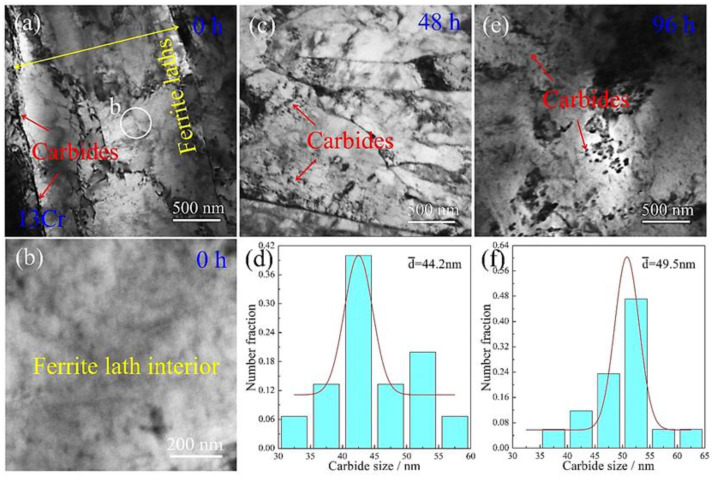
TEM images of the 13Cr samples crept for various durations: (**a**,**b**) 0 h; (**c**,**d**) 48 h and (**e**,**f**) 96 h. (**a**) BF image showing ferrite lath and carbides along the ferrite lath boundary; (**b**) close-up image of the circle in (**a**) showing the morphology inside the ferrite lath interior; (**c**,**e**) BF images of the samples crept for 48 h and 96 h, respectively; (**d**,**f**) corresponding statistical size distribution of carbides.

When the addition of Cr element in the steel was increased up to 13 wt.%, the resulting tempering microstructure was observed (Figure 7a). Similar to in the 1Cr samples, the quenching martensite phase is transformed into a ferrite phase, and the lath morphology is remains vertically aligned after tempering. A portion of ferrite lath boundaries was found to be decorated by carbides (identified in detail in Section 3.5). Inside the ferrite lath interior, several dislocations are left, and no carbide particles can be detected, although the area was examined in a close-up image (Figure 7b). This indicates that the addition of 13 wt.% Cr effectively slows down the formation of carbides, irrespective of whether it occurs along ferrite lath boundaries or inside lath interiors. After the tempered samples suffered from creep deformation, the resulting microstructure was also examined. Figure 7c shows TEM morphology of the 48 h crept samples. The carbide particles appear in ferrite lath interiors, whereas their dimensions are far smaller than those of the 1Cr samples under the same creep conditions. The statistical distribution of the particles obtained from multiple TEM images is shown in Figure 7d. The particle size is in the range of 32–57 nm, with an average size of about 44.2 nm. The microstructure resulting from an increased creep time of 96 h is presented in Figure 7e. A prominent variation in the endurance time is that the particle dimensions become slightly larger. Statistical measurements of the particle size reveal that it ranges from 38 nm to 64 nm, with an average size of 49.5 nm, as shown in Figure 7f. This value is smaller than the size of the alloyed cementite (~67.8 nm) in the 1Cr sample at the same creep time.

### 3.5. Distribution of Chemical Compositions after Creep

**Figure 8:**
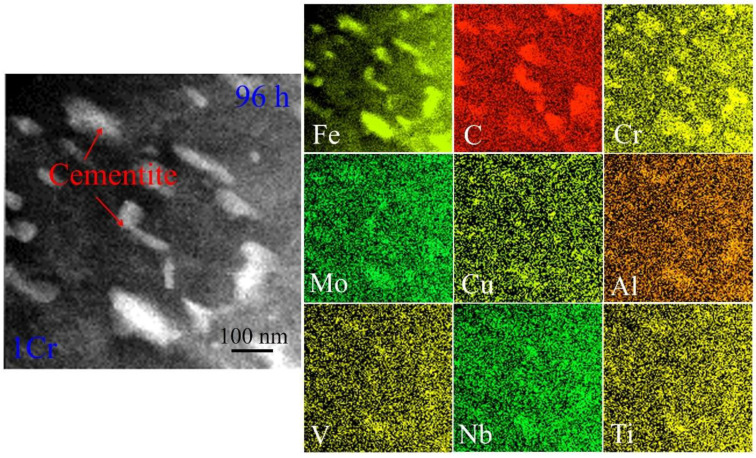
HAADF EDS maps of alloying elements Fe, C, Cr, Mo, Cu, Al, V, Nb and Ti in the 1Cr samples crept for 96 h.

In order to exploit the influence of alloying addition on microstructural evolution, the distribution of chemical compositions in various crept samples was characterized. Figure 8 shows scanning transmission electron microscopy (STEM) EDS maps of the 1Cr samples after creeping for 96 h. The particles embedded in the ferrite matrix are rich in Fe and C atoms, which indicates that the particles still belong to cementite when adding 1 wt.% Cr into the plain carbon steels. As for Cr, element partitioning takes place in the microstructure. Cr atoms are rich in the cementite particles and lean in the ferrite matrix. This indicates that cementite is the preferential site for Cr atoms so that Cr atoms substitute Fe atoms to form the alloyed cementite. The distribution profiles of Fe and C atoms are consistent with the particle morphologies of the cementite in TEM images, whereas this is not the case for Cr atoms. The latter exhibits larger distribution dimension than the particle size. This might mean that Cr atoms are segregated into the cementite from the neighboring ferrite in progress. As for other elements, such as Mo, Cu, Al, V, Nb and Ti, they also tend to segregate into cementite, although the segregation is not clearly demonstrated due to their low contents.

**Figure 9:**
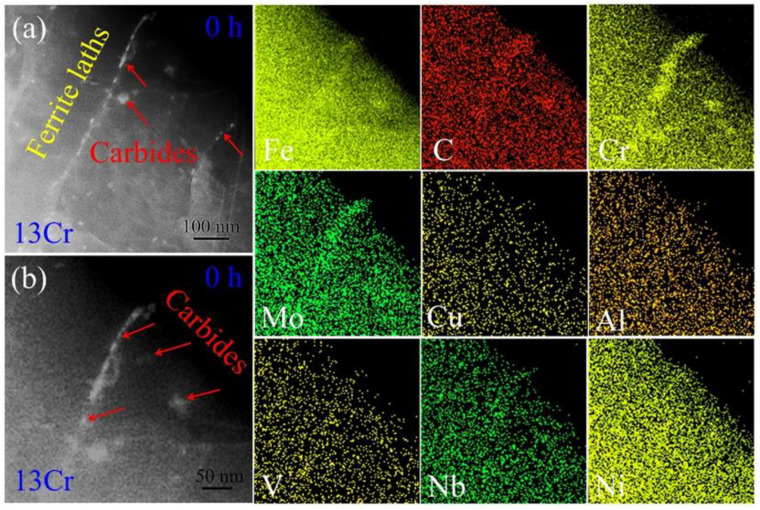
Elemental distribution of the 13Cr creep-free samples: (**a**) HAADF STEM image and (**b**) HAADF EDS images of alloying elements, such as Fe, C, Cr, Mo, Cu, Al, V, Nb and Ni.

Figure 9a is high-angle annular dark-field (HAADF) STEM image of the 13Cr samples without creep deformation. In general, the HAADF image obtained in STEM mode depends on overall atomic mass (Z), and thereby, high atomic masses result in bright contrast in the image (Z-contrast) [30]. In Figure 9a, the carbide particles decorating ferrite lath boundaries exhibit brighter contrast in comparison to the neighboring microstructure. This means that compositional partitioning takes place between ferrite lath boundaries and lath interiors. Furthermore, the distribution of chemical elements was imaged, as shown by STEM EDS maps in Figure 9b. Segregation of Fe and C atoms in the carbides is not obvious anymore; instead, are distributed relatively homogeneously throughout the microstructure. As for Cr, it is rich in the carbides so that an apparent profile of the ferrite lath boundary is formed. These varying distributions indicate that when adding the content of Cr element up to 13 wt.% in plain carbon steels, the martensite is decomposed into high-Cr carbides rather than alloyed cementite upon tempering. Regarding Nb element, it is rich in the carbides, such as in the case of Cr element. As for the remaining trace elements, such as Cu, Al, V, Nb and Ti, no obvious segregation was found in the microstructure. This is associated with their low content in the steels (Table 1).

**Figure 10:**
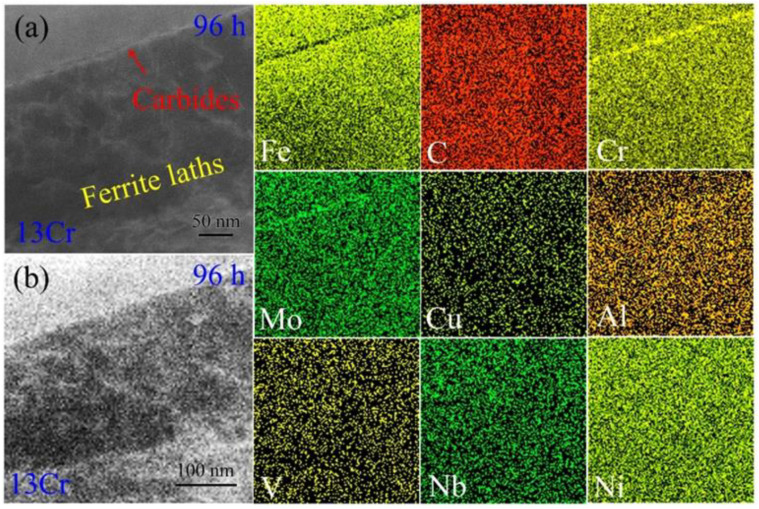
Elemental distribution around ferrite lath boundaries in the 13Cr samples crept for 96 h: (**a**) HAADF STEM image and (**b**) HAADF EDS maps of alloying elements, such as Fe, C, Cr, Mo, Cu, Al, V, Nb and Ni.

After the 13Cr tempered samples were crept for 96 h, the resulting compositional distribution was also analyzed. We first characterized the elemental distribution around ferrite lath boundaries. Figure 10a shows the corresponding HAADF STEM image. The ferrite lath boundary exhibits bright contrast relative to the neighboring microstructure. This indicates that large amounts of heavy atoms are segregated here. Figure 10b shows STEM EDS maps of different elements. It is surprising that Fe atoms are mostly depleted out of the ferrite lath boundary so that a black line is presented in the endurance time. As for C element, there is no obvious segregation along the ferrite lath boundary or inside the lath interiors, which is similar to the initial tempered microstructure in Figure 9b. Regarding Cr element, it is rich along the ferrite lath boundary. Upon careful observation, the aggregation constructs a string of particles like ‘necklaces’ to disperse along the lath boundary. Combined with the distribution features of Fe, C and Cr elements, it can be asserted that Cr atoms are continuously trapped in the carbides at the expense of Fe rejection with prolonged creep time. Similar to Cr element, Mo element is also segregated in the carbides. Additionally, other trace elements, such as Cu, Al, V, Nb and Ni were not observed to be rich in the carbides, perhaps because of their fairly low contents.

**Figure 11:**
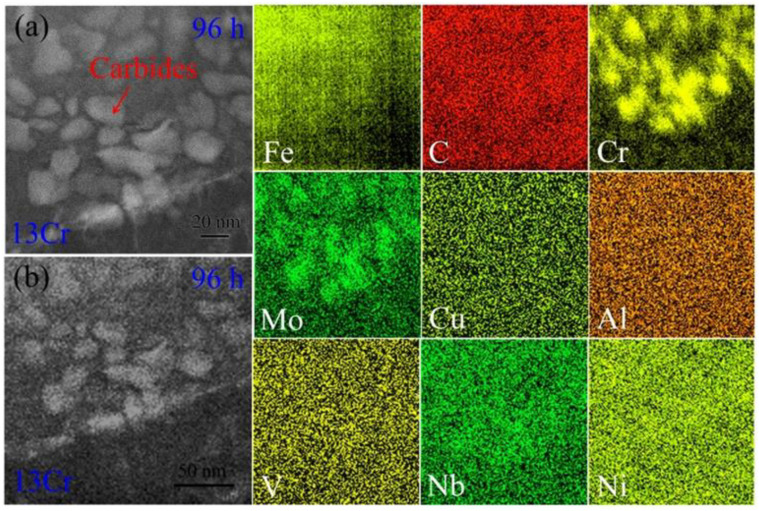
Element distribution of ferrite lath interior in the 13Cr samples crept for 96 h: (**a**) HAADF STEM image and (**b**) HAADF EDS maps of alloying elements, such as Fe, C, Cr, Mo, Cu, Al, V, Nb and Ni.

Figure 11a shows an HAADF STEM image of ferrite lath interior in the 13Cr 96 h crept samples. Compositional partitioning also occurs between the carbides and the neighboring ferrite matrix. The former has a bright contrast, whereas the latter has a dark contrast, indicative of the aggregation of heavy elements in the carbides. Figure 11b is the corresponding STEM EDS maps. Fe atoms are rejected from the carbides, which results in several shadow areas in corresponding positions. C atoms are still found to distribute homogeneously throughout the microstructure. As for Cr element, segregation inside the carbides can be observed in the element map. However, the carbides seem to have a tendency to be aggregated together. This suggests that the carbides initiate growth via the aggregation of several small carbides. Similar to Cr element, the diffusion distribution of Mo atoms is also presented in the element map. As for other trace elements, no obvious segregation is detected in the microstructure due to their much lower contents.

**Figure 12:**
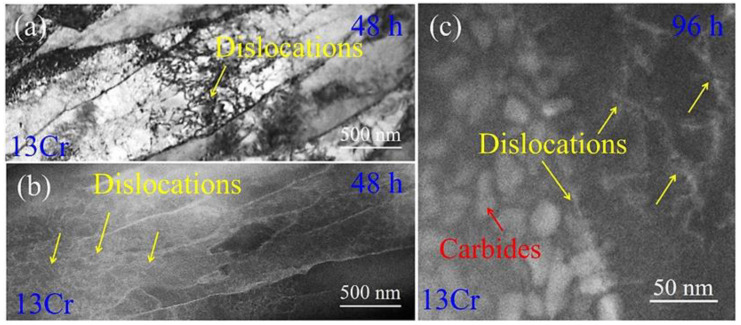
Deformation characterizations around dislocations in the as-crept 13Cr samples: (**a**) TEM BF image showing dislocation tangling in ferrite lath interior after creeping for 48 h and (**b**) HAADF STEM image showing atom segregation on dislocations; (**c**) HAADF STEM image of atom segregation on dislocations in the 96 h crept samples.

Additionally, we also characterized deformation features around dislocation. Dislocation motions are impeded by carbides so that dislocation tangling is formed in the microstructure (Figure 12a). Figure 12b is an HAADF STEM image of the dislocation tangling. It is apparent that dislocation areas generally have bright contrast relative to their vicinities. This distribution feature is more obviously presented in Figure 12c, where a distribution network of Cr element associated with dislocation lines is formed. This result strongly indicates that dislocations provide a pipe for fast elemental diffusion upon creeping.

## 4. Discussion

### 4.1. Preferential Partitioning of Cr to Cementite/Carbides

When the 0Cr samples are austenitized at high temperature, chemical elements distribute homogeneously in the austenite grains due to high diffusion capacity of atoms. The homogeneous compositional distribution is maintained in martensite after water quenching. When the quenching martensite suffers from subsequent tempering, it is decomposed into ferrite and cementite, wherein element partitioning of Fe and C takes place on the basis of the Fe-C phase diagram [31]. Hence, the tempered microstructure is composed of the cementite particles and the ferrite matrix in the 0Cr samples.

It is well known that Cr is a kind of carbide-forming element in terms of carbide chemistry [31,32]. The element is attracted to bond with C to produce carbide. When too little Cr element is added, e.g., 1 wt.% Cr in the plain carbon steels, this content is not sufficient to form carbide with a specific crystal structure. Instead, Cr atoms diffuse into cementite in the substitutional fashion to form alloyed cementite (Fe, Cr)_3_C. Kunitake studied the diffusivity of Cr at 600 °C by fitting a substitutional partitioning model to the experimental measurement of cementite partitioning kinetics in an Fe-0.52C-1.02Mn-0.42Si-1.06Cr-0.22Mo alloy and found the diffusion coefficient of Cr atoms up to 2~4 × 10^−4^ m^2^/s [33]. Such high substitutional diffusivity is beneficial to the formation of alloyed cementite (Fe, Cr)_3_C. Therefore, the tempered microstructure in the 1Cr samples consists of alloyed cementite and a ferrite parent. When the tempered samples are crept, remnant Cr atoms in the ferrite matrix are continuously diffused into the alloyed cementite with the concurrent aid of thermal and mechanical driving. They replace Fe atoms in alloyed cementite interiors, but the intrinsic complex orthorhombic crystal structure of cementite remains unchanged. It has been reported that cementite in Cr-containing steels often has a kinetic advantage over other alloy carbides, and thereby, the transition to more thermodynamically stable alloy carbide from cementite becomes fairly slow and requires long-term diffusion of Cr atoms [32]. The alloyed cementite is thus the dominant form of carbide in the 1Cr crept microstructure. Indeed, the HAADF EDS maps of Fe and Cr elements have demonstrate that Cr atoms become enriched, whereas Fe atoms are still dominant in the alloyed cementite after long-term creep in the 1Cr samples (Figure 8).

When the addition of Cr atoms is increased up to 13 wt.%, the high element content leads to the appearance of a new scenario in the samples. The quenching martensite is decomposed into ferrite and high-Cr carbides, in which the dominant content of Fe and Cr elements is overturned (Figure 9), and the primary element is Cr, but the secondary element is Fe, i.e., the carbide is the type of (Cr, Fe)_23_C_6_ rather than that of (Fe, Cr)_3_C, as above. Corresponding HAADF EDS maps also evidence that Fe and C elements no longer obviously segregate into the carbides in the tempered microstructure (Figure 9). The segregation of Cr element in the (Cr, Fe)_23_C_6_ structure even leads to the rejection of Fe atoms out of the carbides during the creep deformation. In order to achieve thermodynamical stability as much as possible in the particles, remanent Fe atoms are rejected from the carbides, as shown by the shallow areas of the carbide in HAADF EDS maps of Fe element in Figure 10 and Figure 11.

### 4.2. Suppression of Carbide Coarsening

Statistical measurements of cementite dimensions in the 0Cr crept samples manifest that the precipitate size is increased from 55.9 nm in the initial tempering state to 132.5 nm at a long-term creep of 96 h (Figure 4d). It is apparent that cementite particles are coarsened during creep periods. TEM observations coupled with EDS analysis reveal that dislocations exist in the ferrite matrix, and some are pinned by cementite particles (Figure 4e), whereas solutes are segregated on the dislocation lines (Figure 12). This strongly suggests that dislocation motions under a solute atmosphere enhance the coarsening of cementite, i.e., the scavenging effect of dislocations [34,35]. Because Cr element, as the solute, has atomic size misfit with the solvents, a solute atmosphere is formed around dislocations. When the dislocations encounter a growing cementite, solute flow is expected to occur from the atmosphere into the growing precipitate along dislocation cores [35]. This means that once solutes are collected around a moved dislocation, they promote the coarsening of cementite (Figure 4). The accelerated coarsening of precipitates caused by solute diffusivity from moving dislocations has also been demonstrated in Cr-rich precipitates (CRPs) in G115 steel during the creep process [36]. On the other hand, given that dislocation motions are frequently pinned by cementite particles, such a strong obstacle effect makes unpinning very difficult so that the dislocations are developed as sessile dislocations during further creep loading [36]. This dislocation configuration provides an additional pipe diffusion path for Cr atoms from the ferrite matrix to cementite, which also promotes the coarsening of cementite in the 0Cr crept samples.

Our experimental observations have uncovered that a small addition of Cr atoms results in a marked decrease in the coarsening of cementite particles. This means that the Cr element strongly retards cementite coarsening. According to the Lifshitz–Slyozov–Wagner (LSW) theory, the coarsening kinetics of cementite particles can be expressed as a power law relationship [37]:

(1) $$d^{q}-d_{0}^{q}=k_{0}\left(t-t_{0}\right)$$

Furthermore, Equation (1) is sometimes simplified as [37]:

(2) $$d=kt^{n}$$

where *n* = 1/*q*; and *d*_0_ and *d* are the initial and final particle sizes, respectively. Coarsening rate constant, *k*, and exponent, *n*, are the functions of the solubility, boundary energy and diffusion of atoms, wherein *n* is a parameter dependent on the coarsening mechanism of the precipitation particles. When Cr element is doped into the steels, the affinity between Cr and C is stronger than that between Fe and C [37]. Carbon diffusivity is thus decreased due to the degradation of carbon potential in the alloy system [37]. This results in a decrease in the value, *n*, with increased Cr content. The sluggish coarsening caused by the addition of Cr has been recently demonstrated in pearlitic cementite particles, where the value of *n* was 0.246 in Fe-0.8C steel, whereas it was decreased to 0.230 in Fe-0.8C-1Cr steel [37]. Such a suppression effect of Cr doping can also be rationalized on the basis of chemical/mechanical stability of alloyed cementite. The binding energy, *E_binding_*, and formation enthalpy, $\Delta H_{f}$, of the alloyed cementite was deduced using first-principles calculation [38]. As a result, *E_binding_* of Cr-C (~39.61 eV/f.u) was lower than that of Fe-C (~37.62 eV/f.u) [38], indicating that the chemical bond in the former is stronger than that in the latter. Meanwhile, $\Delta H_{f}$ of the alloyed cementite Cr_1_Fe_2_C and Cr_2_Fe_1_C was negative (−0.297~−0.413 eV/f.u), but the value of cementite Fe_3_C was positive (0.143 eV/f.u) [38]. This implies that alloyed cementite forms more easily than cementite (Fe_3_C), and the chemical stability of alloyed cementite is strengthened by Cr doping. Therefore, the coarsening of particles is effectively restrained by the addition of Cr element (Figure 5, Figure 6 and Figure 7).

### 4.3. Enhanced Creep Resistance and Remnant Tensile Strength

It is known that one of the strategies to enhance creep resistance is to introduce precipitate particles into the parent phase, and the resulting precipitation-strengthening effect inhibits deformation strain [8,36,39,40]. For creep at elevated temperatures, the precipitates undergo loading and thermal exposure concurrently. Thermal stability and mechanical stability of the precipitates are two crucial factors for creep strength [39]. The discussion in Section 4.1 and Section 4.2 demonstrates that Cr element is preferentially segregated into cementite, and accordingly, the cementite is transformed to alloyed cementite and, eventually, carbides when Cr content is high enough. These precipitation particles favor structural and chemical stability due to their high binding energy, *E_binding_*, and formation enthalpy, $\Delta H_{f}$ [38]. They maintain the particle dimensions so that the coarsening is slowed down with prolonged creep time. This sluggish effect becomes more apparent with the addition of sufficient Cr atoms into the steels. Hence, dislocation motions are effectively impeded by the thermally stable precipitates on basis of the Orowan mechanism [41,42]. Creep rate correspondingly decreases in the Cr-doped steels (Figure 1).

As to remnant tensile strength in the as-crept samples, it is closely correlated with the evolution of the precipitates during creep periods. The precipitation-strengthening effect is weakened when the cementite particles are coarsened, and the resulting remnant tensile strength is degraded with the progressive creep time in the 0Cr sample. However, the addition of Cr element delays the coarsening kinetics of the precipitates. This variation largely postpones the degradation of remnant tensile strength, as shown by the nearly identical strength during the entire duration periods in the 1Cr samples (Figure 2). Furthermore, when Cr element is added up to 13 wt.%, the precipitation response is altered, and thereby, the alloyed cementite is replaced by high-Cr carbides (Figure 9). The carbides are slowly precipitated from the ferrite matrix, as opposed to continuous coarsening in the 0Cr samples. This sluggish precipitation in turn results in an increasingly significant precipitation-strengthening effect. Hence, remnant tensile strength is continuously increased with prolonged creep time in the 13Cr samples (Figure 2).

## 5. Conclusions

In this study, two types of novel alloying steels with the addition of Cr element were designed relative to the original plain carbon steel, which is always used as oil casing. After interrupted creep testing was performed for various endurance periods, the resulting remnant tensile strength was investigated. The corresponding microstructures and elemental partitioning were also characterized using TEM microscopy and HAADF mapping. The main findings are drawn as follows:

(1) Unlike the degraded remnant tensile strength in the 0Cr samples, prior creep history in the 1Cr samples enables subsequent tensile yield strength of around ~940 MPa without obvious degradation. When the addition of Cr element is further increased to 13 wt.%, prior creep history enhances remnant tensile strength from ~840 MPa to ~960 MPa with prolonged creep time from 0 h to 96 h.
(2) The coarsening of cementite particles is remarkably retarded in the 1Cr samples after creep in comparison to that in the 0Cr samples. For the 13Cr samples, however, the microstructure undergoes precipitation of carbide particles without the occurrence of coarsening due to a much slower precipitation response.
(3) Cr element is prone to be segregated into the precipitate particles. The cementite in the 0Cr samples is evolved to alloyed cementite when 1 wt.% Cr is added, whereas carbide particles are formed when the addition content is increased to 13 wt.%. The intrinsic thermal stability possessed by both alloyed cementite and carbides continuously favors a precipitation-strength effect, which synchronously slows down the creep rate and enhances remnant tensile strength.

## Figures and Tables

**Table 1:** Actual chemical compositions (wt.%) of three types of steels used in this study.

	C	Si	Mn	P	S	Cr	Mo	Ni	Nb V Ti	Cu	Al
0Cr	0.26	0.24	1.27	0.015	0.0018	0.042	0.008	0.020	≤0.0079	0.053	0.016
1Cr	0.17	0.24	0.98	0.011	0.0034	0.99	0.33	0.059	0.019/0.029/0.013	0.21	0.017
13Cr	0.26	0.19	0.17	0.011	0.0002	12.4	2.04	5.6	0.0001/0.013/0.0002	0.06	0.033

## Data Availability

The data that support the findings of this study are available from the corresponding author upon reasonable request.
